# Expression and Functional Analysis of the Metallothionein and Metal-Responsive Transcription Factor 1 in *Phascolosoma esculenta* under Zn Stress

**DOI:** 10.3390/ijms25137368

**Published:** 2024-07-05

**Authors:** Shenwei Gu, Jingqian Wang, Xinming Gao, Xuebin Zheng, Yang Liu, Yiner Chen, Lianlian Sun, Junquan Zhu

**Affiliations:** 1 Key Laboratory of Aquacultural Biotechnology, Ministry of Education, Ningbo University, Ningbo 315211, Chinachenyiner@nbu.edu.cn (Y.C.); 2 Key Laboratory of Marine Biotechnology of Zhejiang Province, College of Marine Sciences, Ningbo University, Ningbo 315211, China; 3 College of Ecology, Lishui University, Lishui 323000, China

**Keywords:** *Phascolosoma esculenta*, metallothionein, metal-responsive transcription factor 1, zinc, intestine

## Abstract

Metallothioneins (MTs) are non-enzymatic metal-binding proteins widely found in animals, plants, and microorganisms and are regulated by metal-responsive transcription factor 1 (MTF1). MT and MTF1 play crucial roles in detoxification, antioxidation, and anti-apoptosis. Therefore, they are key factors allowing organisms to endure the toxicity of heavy metal pollution. *Phascolosoma esculenta* is a marine invertebrate that inhabits intertidal zones and has a high tolerance to heavy metal stress. In this study, we cloned and identified *MT* and *MTF1* genes from *P. esculenta* (designated as *PeMT* and *PeMTF1*). *PeMT* and *PeMTF1* were widely expressed in all tissues and highly expressed in the intestine. When exposed to 16.8, 33.6, and 84 mg/L of zinc ions, the expression levels of *PeMT* and *PeMTF1* in the intestine increased first and then decreased, peaking at 12 and 6 h, respectively, indicating that both *PeMT* and *PeMTF1* rapidly responded to Zn stress. The recombinant pGEX-6p-1-MT protein enhanced the Zn tolerance of *Escherichia coli* and showed a dose-dependent ABTS free radical scavenging ability. After RNA interference (RNAi) with *PeMT* and 24 h of Zn stress, the oxidative stress indices (MDA content, SOD activity, and GSH content) and the apoptosis indices (Caspase 3, Caspase 8, and Caspase 9 activities) were significantly increased, implying that *Pe*MT plays an important role in Zn detoxification, antioxidation, and anti-apoptosis. Moreover, the expression level of *PeMT* in the intestine was significantly decreased after RNAi with *PeMTF1* and 24 h of Zn stress, which preliminarily proved that *Pe*MTF1 has a regulatory effect on *Pe*MT. Our data suggest that *Pe*MT and *Pe*MTF1 play important roles in the resistance of *P. esculenta* to Zn stress and are the key factors allowing *P. esculenta* to endure the toxicity of Zn.

## 1. Introduction

Heavy metal pollution is a global environmental problem [1]. Human activities have increased the concentrations of heavy metals in water [2,3], which then accumulate in aquatic organisms through respiration, feeding, and skin penetration [4]. Among heavy metals, zinc (Zn) is an essential trace element for organisms that participates in DNA replication, transcription, cell proliferation, and a variety of metabolic processes [5]. However, excessive levels of Zn are harmful to aquatic organisms. Zn affects the stability of the lipid bilayer, thereby destroying the structure of enzymes and affecting the active transportation of cell membranes [6]. In addition, it inhibits the absorption of other trace elements, resulting in anemia and enzyme inactivation [7]. Moreover, studies have shown that excessive Zn causes oxidative stress, resulting in an imbalance of reactive oxygen species [8], and influences apoptosis by acting on several molecular regulators of programmed cell death, including caspases and proteins from the Bcl and Bax families [9].

Metallothioneins (MTs) are non-enzymatic metal-binding proteins widely found in animals, plants, and microorganisms. They are the first line allowing organisms to endure the toxicity of heavy metals [10,11] and have the characteristics of low molecular weight and high cysteine (Cys) content [12]. Their Cys residues can strongly chelate heavy metals and aid in detoxification [13,14], and the sulfhydryl group exists in a reduced state, allowing them to react with free radicals and act as antioxidants [15]. Additionally, MTs play important roles in anti-apoptosis; high expression levels of MTs can enhance the anti-apoptosis ability of organisms, whereas low expression levels of MTs may promote apoptosis [16]. The expression of MTs is regulated by metal-responsive transcription factor 1 (MTF1). As a zinc-induced transcription factor, MTF1 is activated by Zn^2+^ and regulates MT by binding to metal-responsive elements (MREs) in the promoter of MT [17]. MT and MTF1 play important roles in the regulation of Zn homeostasis, which protects the body from external stimuli and helps maintain normal physiological functions. Studies have shown that vertebrate MT and MTF1 homologs are highly conserved [18], whereas invertebrate MT and MTF1 homologs have low homology, and their functions require further exploration [19].

*Phascolosoma esculenta* is a valuable marine invertebrate belonging to the phylum Sipuncula [20]. It inhabits intertidal zones and feeds on organic debris and benthic diatoms [21], which makes it vulnerable to heavy metal pollution. Previous studies have shown that *P. esculenta* has a high tolerance to heavy metal stress [22]; however, there is still a lack of research on the adaptation mechanism of *P. esculenta* in response to heavy metals, and whether *Pe*MT and *Pe*MTF1 are involved in the resistance to heavy metals has not been reported.

Therefore, in this study, we cloned and identified the *PeMT* and *PeMTF1* genes and detected the tissue-specific expression of *PeMT* and *PeMTF1* mRNA, and their temporal expression profiles under Zn stress. We then assessed the Zn tolerance of *E. coli* overexpressing pGEX-6p-1-MT and the in vitro antioxidant activity of the recombinant pGEX-6p-1-MT protein. Additionally, RNA interference (RNAi) and Zn stress were performed at the individual level, and changes in intestinal oxidative stress and apoptosis indices after interference with *PeMT*, as well as changes in intestinal *PeMT* mRNA expression after interference with *PeMTF1*, were detected. This study fills the gap in functional research on MT and MTF1 in Sipuncula and provides basic data for studying the molecular toxicology of *P. esculenta*.

## 2. Results

### 2.1. Sequence Features and Bioinformatics Analysis of PeMT

**Figure 1:**
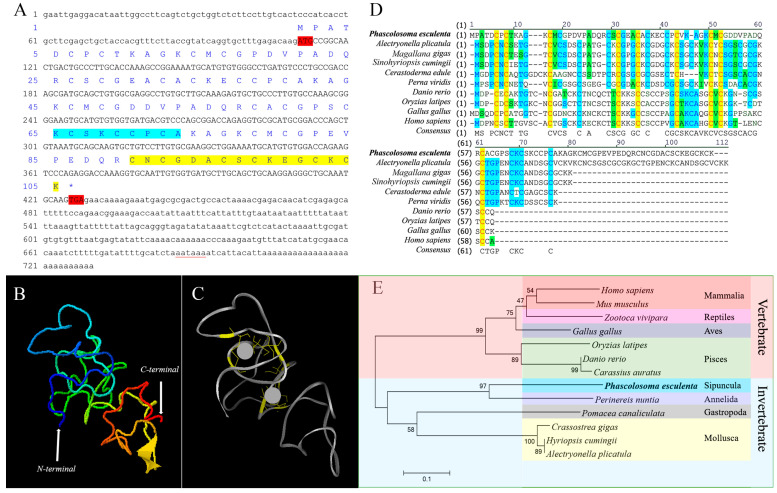
Full-length cloning and bioinformatic analysis of *Pe*MT. (**A**) Completed cDNA and deduced amino acid sequence of *Pe*MT. The red font is the start codon and the end codon. The red underline is a putative polyadenylation signal. The blue shadow is the characteristic sequence of invertebrate MTs. The yellow shadow is similar to the characteristic sequence of Mollusca MTs. * represents the termination codon. (**B**) N-terminus and C-terminus of *Pe*MT. (**C**) Yellow parts show the conserved Cys residues, and gray spheres indicate Zn^2+^. (**D**) Multiple sequence alignment of *Pe*MT. The similarities between *Pe*MT and its homologs in *A. plicatula*, *C. gigas*, *S. cumingii*, *C. edule*, *P. viridis*, *D. rerio*, *O. latipes*, *M. gallus*, and *H. sapiens* were 37.1%, 36.8%, 33.3%, 35.2%, 32.4%, 25.7%, 20.0%, 17.1%, and 16.2%, respectively. The same amino acid residues are shaded in yellow, blue regions indicate amino acid residues with a similarity greater than 50%, and green regions represent lower similarity. (**E**) Phylogenetic analysis of MT homologous proteins. *P. esculenta* is shown in bold font, and *Pe*MT belongs to the invertebrate branch.

The full-length cDNA of *PeMT* was 730 bp in length (GenBank Accession No. QQ848618.1), containing a 110 bp 5′-untranslated region (UTR), a 318 bp open reading frame (ORF) that encoded 105 amino acids, and a 302 bp 3′-UTR with a conserved polyadenylation signal (AATAAA). The predicted molecular weight was 10.7 kDa, and the isoelectric point was 7.63. The “CKCSKCCPCA” was consistent with the conserved motif “CKCXXXCXCX” of invertebrate MTs and the “CNCGDACSCKEGCKCK” in the C-terminus was similar to the conserved motif “CXCXXXCTGXXXCXCXXXCXCK” of Mollusca MTs. This protein contains 28 Cys and 12 lysine (Lys) residues, and no histidine (His) or aromatic amino acids (Phe, Trp, Tyr). The 28 Cys residues were mainly presented in the forms of Cys-Cys, Cys-X-Cys, Cys-X-X-Cys, and Cys-X-X-X-Cys (Figure 1A). Among them, nine conserved Cys residues were predicted to bind to two Zn^2+^ units (Figure 1B,C). The similarities between *Pe*MT and its homologs in *Alectryonella plicatula*, *Magallana gigas*, *Sinohyriopsis cumingii*, *Cerastoderma edule*, *Perna viridis*, *Gallus gallus*, *Oryzias latipes*, *Danio rerio*, and *Homo sapiens* were 37.1%, 36.8%, 33.3%, 35.2%, 32.4%, 25.7%, 20.0%, 17.1%, and 16.2%, respectively (Figure 1D), indicating that *Pe*MT has low homology with other homologs. Phylogenetic tree analysis revealed that the MT homologs were divided into invertebrate and vertebrate branches. *Pe*MT clustered in the invertebrate branch and had the closest relationship with MT from *Perinereis nuntia* among the selected species (Figure 1E).

### 2.2. Sequence Features and Bioinformatics Analysis of PeMTF1

**Figure 2:**
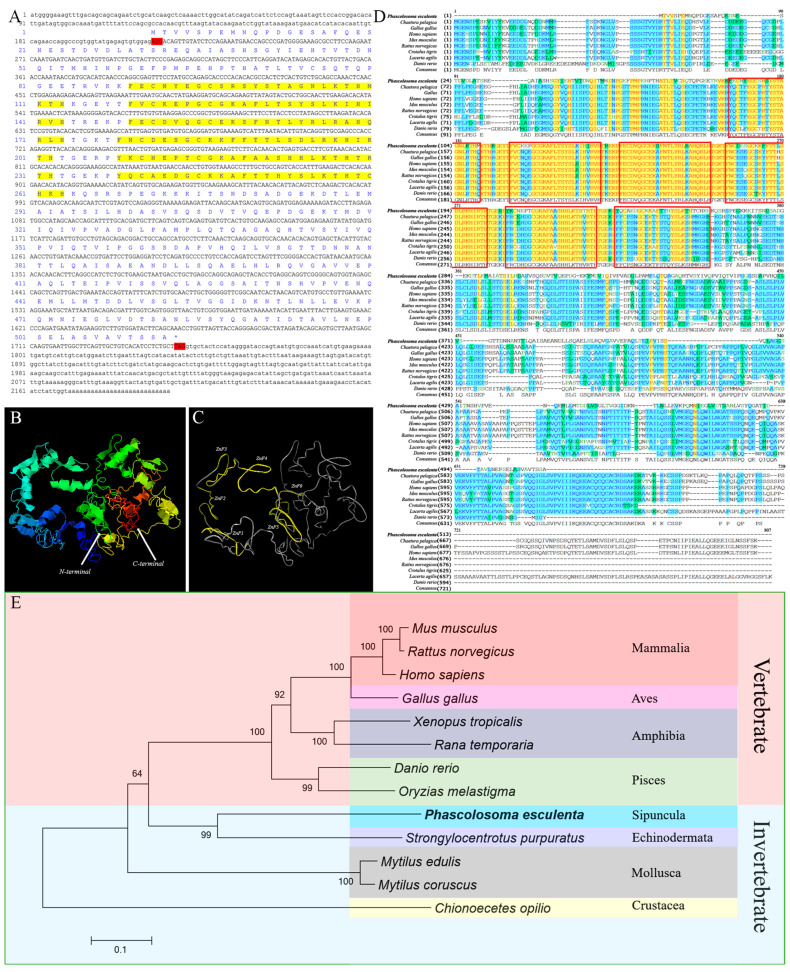
Full-length cloning and bioinformatic analysis of *Pe*MTF1. (**A**) Completed cDNA and deduced amino acid sequence of *Pe*MTF1. The red shadow is the start codon and the end codon. The yellow shadows are the C_2_H_2_-type zinc finger domains. * represents the termination codon. (**B**) N-terminus and C-terminus of the protein sequence. (**C**) Yellow parts are the conserved C_2_H_2_-type zinc finger domains. (**D**) Multiple sequence alignment of *Pe*MTF1. The similarities between *Pe*MTF1 and its homologs in *C. pelagica*, *G. gallus*, *H. sapiens*, *M. musculus*, *R. norvegicus*, *C. tigris*, *L. agilis*, and *D. rerio* were 36.4%, 37.0%, 34.4%, 37.0%, 38.7%, 39.9%, 36.5%, and 43.0%, respectively, and the similarities of the zinc finger domains were 84.0%, 84.0%, 84.6%, 84.6%, 84.6%, 84.6%, 84.6%, and 85.7%, respectively. The red boxes are six conserved C_2_H_2_-type zinc finger domains. The same amino acid residues are shaded in yellow, blue regions indicate amino acid residues with a similarity greater than 50%, and green regions represent lower similarity. (**E**) Phylogenetic analysis of MTF1 homologous proteins. *P. esculenta* is shown in bold font, and *Pe*MTF1 belongs to the invertebrate branch.

The full-length cDNA of *PeMTF1* was 2197 bp in length (GenBank Accession No. QQ848619.1), containing a 212 bp 5′-UTR, a 1539 bp ORF that encoded 512 amino acids, and a 446 bp 3′-UTR (Figure 2A). The predicted molecular weight was 55.74 kDa, and the isoelectric point was 5.83. Moreover, *Pe*MTF1 was predicted to be localized in the cytoplasm and nucleus. The N-terminus of *Pe*MTF1 contains six conserved C_2_H_2_-type zinc finger domains (Figure 2B,C). The similarities between *Pe*MTF1 and its homologs in *Chaetura pelagica*, *G. gallus*, *H. sapiens*, *Mus musculus*, *Rattus norvegicus*, *Crotalus tigris*, *Lacerta agilis*, and *D. rerio* were 36.4%, 37.0%, 34.4%, 37.0%, 38.7%, 39.9%, 36.5%, and 43.0%, respectively. However, the similarities between their zinc finger domains were 84.0%, 84.0%, 84.6%, 84.6%, 84.6%, 84.6%, 84.6%, and 85.7%, respectively (Figure 2D), indicating that *Pe*MTF1 has low homology with the other homologs, but the zinc finger domains are highly conserved. Phylogenetic tree analysis showed that the MTF1 homologs were divided into invertebrate and vertebrate branches. *Pe*MTF1 clustered in the invertebrate branch and had the closest relationship with MTF1 from *Strongylocentrotus purpuratus* among the selected species (Figure 2E).

### 2.3. Tissue-Specific Expression of PeMT and PeMTF1 mRNA

**Figure 3:**
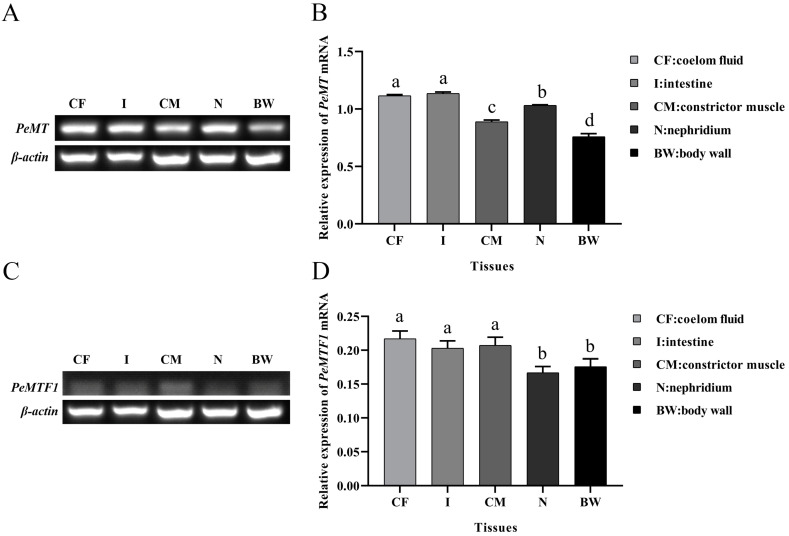
Tissue-specific expression of *PeMT* and *PeMTF1* mRNA. (**A**) RT-PCR detection of *PeMT* mRNA. (**B**) Gray value of RT-PCR analyzed using Image J. (**C**) RT-PCR detection of *PeMTF1* mRNA. (**D**) Gray value of RT-PCR analyzed using Image J. *β-actin* gene was used as an internal reference, and all data are presented as the mean ± SD (*n* = 3). Different letters indicate significant differences among the tissues (*p* < 0.05).

The relative expression levels of *PeMT* and *PeMTF1* mRNA in the coelom fluid, intestine, constrictor muscle, nephridium, and body wall were detected using RT-PCR. The results showed that *PeMT* and *PeMTF1* mRNA were widely expressed in all tissues. *PeMT* mRNA had the highest expression level in the intestine, followed by the coelom fluid, nephridium, constrictor muscle, and body wall (Figure 3A,B), whereas *PeMTF1* mRNA showed the higher expression levels in the coelom fluid, constrictor muscle, and intestine than in the body wall and nephridium (Figure 3C,D).

### 2.4. Expression Changes of PeMT and PeMTF1 mRNA in the Intestine under Zn Stress

**Figure 4:**
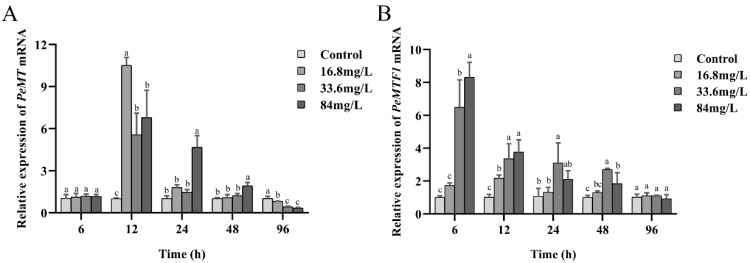
Expression changes of (**A**) *PeMT* and (**B**) *PeMTF1* mRNA in the intestine of *P. esculenta* under Zn stress. *β-actin* gene was used as an internal reference, and all data are presented as the mean ± SD (*n* = 3). Different letters indicate significant differences among the groups (*p* < 0.05).

The temporal expression profiles of *PeMT* and *PeMTF1* mRNA in the intestine of *P. esculenta*, following exposure to different concentrations of Zn, are shown in Figure 4. No significant differences were observed in the control group (*p* > 0.05). However, they were upregulated first and then downregulated under Zn stress and reached the maximum at 12 and 6 h, respectively. When stressed for 24 and 48 h, the expression level of *PeMT* mRNA in the 84 mg/L group was significantly higher than that in the other groups (*p* < 0.05). After 96 h of Zn stress, the expression level of *PeMT* mRNA in the Zn stress groups was significantly lower than that in the control group (*p* < 0.05). Additionally, the expression level of *PeMTF1* mRNA in the Zn stress groups was higher than that in the control group at all time points (except at 96 h of exposure to 84 mg/L Zn^2+^).

### 2.5. Prokaryotic Expression and Protein Purification of pGEX-6p-1-MT

**Figure 5:**
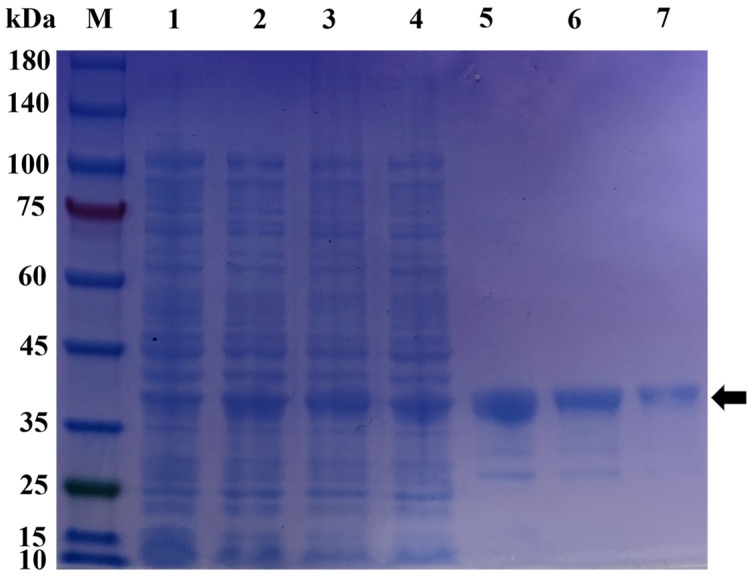
Expression and purification of the recombinant pGEX-6p-1-MT. Line M: marker; Line 1 shows the cell lysate of pGEX-6p-1-MT without induction; Line 2 shows the cell lysate of pGEX-6p-1-MT induced by 1 mM IPTG (isopropyl-β-D-thiogalactoside); Line 3 shows the supernatant of cell lysate; Line 4 shows the precipitation of cell lysate; Lines 5–7 show the purified protein; black arrow: the expressed and purified protein.

The recombinant pGEX-6p-1-MT protein was obtained using a prokaryotic expression system. SDS-PAGE (sodium dodecyl sulfate–polyacrylamide gel electrophoresis) analysis showed that it was expressed in the supernatant and precipitate of broken *E. coli* cells. Then, the supernatant was purified, and a single band with a molecular weight of 36.7 kDa was obtained, which was consistent with the theoretical value predicted by the Expasy ProtParam tool (Figure 5). The concentration of the purified pGEX-6p-1-MT protein was 1.44 mg/mL.

### 2.6. Zn Tolerance of the Recombinant E. coli

**Figure 6:**
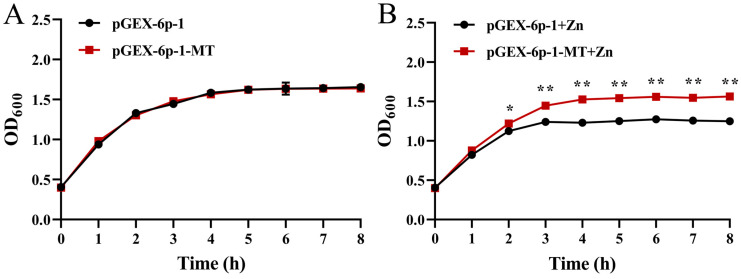
The growth status of the bacteria that transformed pGEX-6p-1-MT and pGEX-6p-1: (**A**) the control group; (**B**) the 0.3 mM Zn stress group. All data are presented as the mean ± SD (*n* = 3). “*”: *p* < 0.05; “**”: *p* < 0.01.

As shown in Figure 6, the growth rates of the bacteria that transformed pGEX-6p-1-MT and pGEX-6p-1 were almost the same in the control group, whereas they were inhibited by 0.3 mM Zn stress. The OD_600_ value of the recombinant strain was significantly higher than that of the control strain after 2 h of Zn stress (*p* < 0.05) and reached an extremely significant level after 3 h of Zn stress (*p* < 0.01).

### 2.7. Antioxidant Capacity of the Recombinant pGEX-6p-1-MT

**Figure 7:**
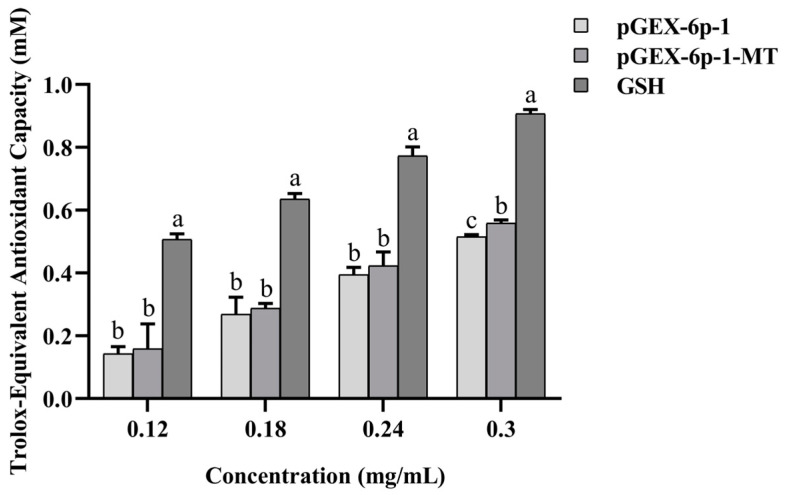
The ABTS free radical scavenging ability of the recombinant *Pe*MT. Different concentrations of pGEX-6p-1 and GSH were set as the control groups. All data are presented as the mean ± SD (*n* = 3); different letters indicate significant differences among the groups (*p* < 0.05).

The ABTS radical scavenging abilities of GSH, pGEX-6p-1-MT, and pGEX-6p-1 were positively correlated with their concentrations and were in the following order: GSH > pGEX-6p-1-MT > pGEX-6p-1. At concentrations of 0.12, 0.18, and 0.24 mg/mL, no significant differences were observed between pGEX-6p-1-MT and pGEX-6p-1 (*p* > 0.05). However, at 0.3 mg/L, the ABTS radical scavenging ability of pGEX-6p-1-MT was significantly higher than that of pGEX-6p-1 (Figure 7).

### 2.8. Changes in Intestinal Oxidative Stress and Apoptosis Indices after RNAi with PeMT and Zn Stress

**Figure 8:**
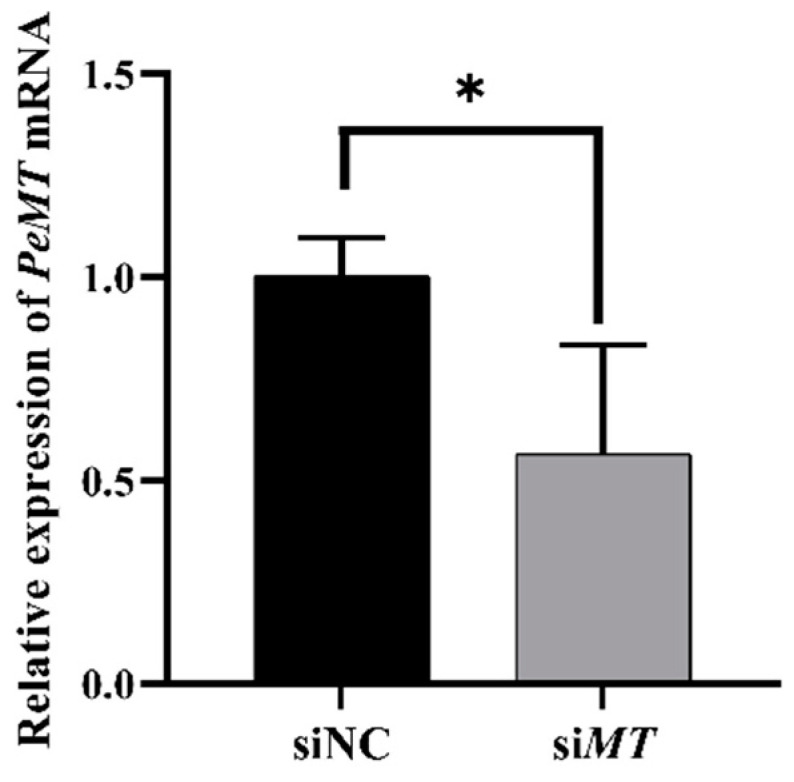
Changes in the relative expression of *PeMT* mRNA after RNAi with *PeMT* and 24 h of Zn stress. *β-actin* gene was used as an internal reference, and all data are presented as the mean ± SD (*n* = 4), “*”: *p* < 0.05.

**Figure 9:**
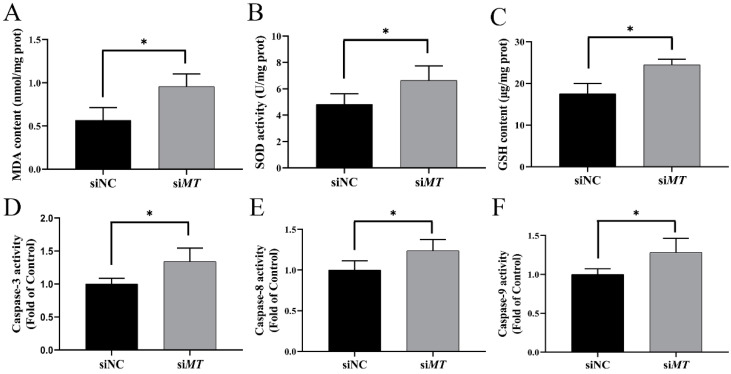
Changes in oxidative stress and apoptosis indices after RNAi with *PeMT* and 24 h of Zn stress. (**A**) MDA content, (**B**) SOD activity, (**C**) GSH content, (**D**) Caspase 3 activity, (**E**) Caspase 8 activity, and (**F**) Caspase 9 activity. All data are presented as the mean ± SD (*n* = 4), “*”: *p* < 0.05.

Compared to the siNC group, the expression level of intestinal *PeMT* mRNA in the si*MT* group was significantly decreased by 46.89% after RNAi and 24 h of Zn stress (*p* < 0.05) (Figure 8). At the same time, the malondialdehyde (MDA) content, superoxide dismutase (SOD) activity, and glutathione (GSH) content were significantly increased to 169%, 137.76%, and 139.62% of the siNC group, respectively (*p* < 0.05) (Figure 9A–C). Moreover, the Caspase 3, Caspase 8, and Caspase 9 activities were significantly increased to 134.14%, 123.64%, and 128.13% of the siNC group, respectively (*p* < 0.05) (Figure 9D–F).

### 2.9. Changes in Intestinal PeMT mRNA Expression Level after RNAi with PeMTF1 and Zn Stress

**Figure 10:**
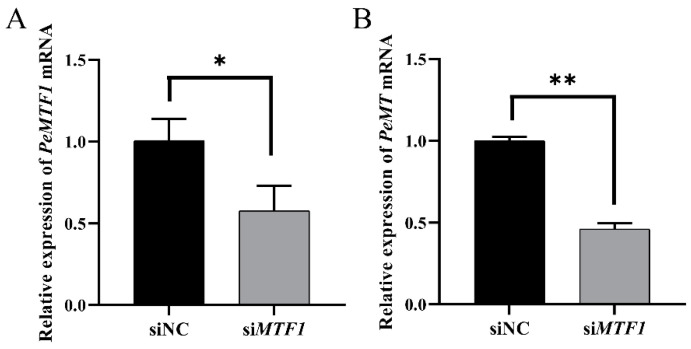
The expression changes of (**A**) *PeMTF1* and (**B**) *PeMT* mRNA in the intestine of *P. esculenta* after RNAi with *PeMTF1* and 24 h of Zn stress. *β-actin* gene was used as an internal reference, and all data are presented as the mean ± SD (*n* = 4), “*”: *p* < 0.05, “**”: *p* < 0.01.

Compared to the siNC group, the expression level of intestinal *PeMTF1* mRNA in the si*MTF1* group was significantly decreased by 42.69% (*p* < 0.05) (Figure 10A), and the expression level of intestinal *PeMT* mRNA decreased by 53.95% (*p* < 0.01) (Figure 10B).

## 3. Discussion

### 3.1. Sequence Features and Protein Structure of PeMT and PeMTF1

The full-length *PeMT* cDNA was cloned from *P. esculenta*. The “CKCSKCCPCA” was in line with the conserved motif “CKCXXXCXCX” of invertebrate MTs, and the “CNCGDACSCKEGCKCK” in the C-terminus was similar to the conserved motif “CXCXXXCTGXXXCXCXXXCXCK” of Mollusca MTs [23]. Moreover, the conserved polyadenylation signal “AATAAA” was observed in the 3′-UTR, which is crucial for mRNA localization and translation [24] and has been found in the MTs of Mollusca [25], Crustacea [26], and Pisces [27]. *Pe*MT showed low similarity to other homologs, and similar results have been found in invertebrate MTs of *Hermetia illucens* [28], *Oxya chinensis* [29], and *Apostichopus japonicus* [30]. *Pe*MT contains 28 Cys and 12 Lys residues. These Cys residues exist in the motifs of Cys-Cys, Cys-X-Cys, Cys-X-X-Cys, and Cys-X-X-X-Cys, which show great affinity for binding heavy metals such as Zn, Cu, and Cd, and participate in divalent detoxification and maintain the stability of metal binding clusters together with Lys [25,31,32]. Studies have shown that the tertiary structure of vertebrate MTs is conserved, consisting of an *α* domain and a *β* domain, which is dumbbell-shaped [33]. However, it varies among invertebrate MTs. For example, *Charybdis japonica* and *Portunus trituberculatus* [31,32] MTs consist of two *β* domains. The *Crassostrea gigas* MT1 consists of an *α* domain and a *β* domain, and the *C. gigas* MT2 consists of an *α* domain and two *β* domains [34]. Moreover, the *Drosophila melanogaster* MT has only one domain [35]. In this study, *Pe*MT was also composed of one domain, further proving that MTs convergently evolved.

Additionally, the full-length *PeMTF1* cDNA was cloned. *Pe*MTF1 was predicted to be located in the cytoplasm and nucleus. Previous studies have shown that MTF1 is located in the cytoplasm and nucleus under normal conditions. However, when cells are stimulated by heavy metals, hypoxia, and other external stimuli. MTF1 may translocate to the nucleus and bind to serval target genes [17]. Moreover, sequence analysis showed that the N-terminus of *Pe*MTF1 has six conserved C_2_H_2_-type zinc finger domains that have high homology with other homologs. The zinc finger domains play important roles in metal regulation and DNA binding [36], and their high conservation indicates that they may have conserved functions. However, in other regions, *Pe*MTF1 showed low similarity to other homologs and lacked transactivation domains. In a study by Ren et al. [37], MTF1 homologs of *Cerapachys biroi*, *Hymenoptera*, and *Oxya chinesis* showed similar characteristics. In addition, Ferencz et al. [38] found that the splicer MTF1-1.1a of *Cyprinus carpio* has six zinc finger domains and a truncated acidic domain but lacks proline and serine/threonine domains. Based on these results, we identified *Pe*MTF1 as a new member of the MTF1 superfamily.

### 3.2. Tissue-Specific Expression of PeMT and PeMTF1 and Their Expression Changes under Zn Stress

Extensive studies have reported that *MT* is widely expressed in all vertebrate and invertebrate tissues [39], and tissues with high expression levels of *MT* were determined to be different among species. For example, *Meretrix meretrix* [40], *Haliotis discus hannai* [41], and *Litopenaeus vannamei* [42] showed the highest *MT* expression levels in the liver, while MTs of liverless species such as *A. japonicus* [30], *H. illucens* [28], and *O. chinensis* [29] were highly expressed in the intestine. Consistent with these findings, *PeMT* was widely expressed in the tested tissues, with the intestine showing the highest expression. The intestine is one of the main organs involved in nutrient absorption and immune defense [43,44], and high expression of *PeMT* indicates that *Pe*MT plays an important role in the intestine of *P. esculenta*. Therefore, we further analyzed the temporal expression profile of intestinal *PeMT* under four concentrations (0, 16.8, 33.6, and 84 mg/L) of Zn^2+^. The expression pattern of *PeMT* showed an inverted “U” shape as the stress time increased, which is consistent with previous studies [45]. For instance, when exposed to Cd and Zn, the expression level of *MT* in the gills of *Septifer virgatus* increased significantly and reached a maximum after 5 days [46]. In *C. japonica*, the expression level of *MT* significantly increased within 12 h of Pb stress and then slowly decreased [47]. Additionally, *A. japonicus MT* displayed the same trend under Cd and Zn stress [30]. The increase in *MTs* in the early stages indicates that *MTs* rapidly respond to heavy metal stress [46], whereas the decrease in the later stages may be related to peroxidation stress. These findings confirm our speculation that *PeMT* is a functional gene involved in Zn detoxification in *P. esculenta* and is expected to be a biomarker for monitoring Zn pollution.

*PeMTF1* was also expressed in the tested tissues, and its expression levels in the coelom fluid, constrictor muscle, and intestine were higher than those in the body wall and nephridium. Under Zn stress, it showed the same trend as *PeMT* and reached the maximum at 6 h. In *C. carpio*, the expression of *MTF1-1.1a* was first upregulated and then downregulated under Cd stress, reaching a maximum at 48 h [38]. In the liver of *Pelteobagrus fulvidraco*, the expression levels of *MTF1* and *MT* significantly increased within 48 h of Zn stress [17]. In *C. gigas*, the expression levels of *MTF1* and *MT* were induced under 0.1 ppm Cd, and the response of *MTF1* was faster than that of *MT* [48]. These findings indicate that MTF1 plays an important role in the response to oxidative stress induced by heavy metals, and its response is faster than that of MT, which may be involved in the regulation of MT.

### 3.3. Detoxification, Antioxidant, and Anti-Apoptosis Functions of PeMT

MTs play an important role in heavy metal detoxification and increase metal tolerance in organisms [49]. Wang et al. [50] inserted *Sinopotamon MT* into the pGEX-4t-1 vector and exposed it to varying concentrations of Cd, Cu, and Zn. It was found that the recombinant strain showed stronger metal tolerance than the control group. Yang et al. [40] constructed the recombinant pET32a-MnMT and found that it could bind to Cu^2+^ and Cd^2+^, making the recombinant strain more resistant to toxic heavy metals. Li et al. [51] constructed the recombinant pET28a-NtMT2F and found that the growth rate of the recombinant strain was faster than that of the control group when exposed to Cd. In the present study, we induced the expression of *Pe*MT in *E. coli* and exposed the cells to Zn^2+^. The results showed that Zn^2+^ inhibited the growth of *E. coli*, but the growth of the recombinant strain was better than that of the control. Based on these results, we believe that *Pe*MT exerts detoxifying effects and enhances Zn tolerance in *E. coli*.

The sulfhydryl group of MTs exists in a reduced state and can react with free radicals to exert an antioxidant function [52,53]. Recombinant human MT-III has been reported to reduce the oxidative damage caused by Cu and Cd stress [54]. Purified *Channa punctate* MT has been shown to have in vitro activities; it plays an important role in scavenging superoxide radicals (O^2−^), ABTS radicals, and DPPH radicals and can protect the body from oxidative damage induced by Fe-NTA [55]. In this study, the recombinant pGEX-6p-1-MT protein was used to conduct an in vitro experiment. The results showed that *Pe*MT exhibited ABTS radical scavenging ability in a dose-dependent manner. These findings indicated that *Pe*MT has antioxidant functions and plays an important role in resisting Zn-induced oxidative stress.

To further investigate the functions of *Pe*MT, we performed RNAi at the individual level. After RNAi with *PeMT* and 24 h of Zn stress, the expression level of *PeMT* was significantly decreased by 46.89%, and the oxidative indices (MDA, SOD, and GSH) and apoptosis indices (Caspase 3, Caspase 8, and Caspase 9) were significantly increased. MDA is the product of lipid peroxidation, and its content is related to the degree of oxidative damage [56]. SOD is the first barrier of organisms’ antioxidant defense, and it can disproportionate the superoxide anion (O^2−^) to H_2_O_2_ and O_2_ [57]. GSH acts as the substrate of GSH-Px and is involved in the removal of H_2_O_2_ [58]. Additionally, when oxidative stress exceeds physiological tolerance, Caspase 8 and Caspase 9 can bind to apoptotic signals, thus activating Caspase 3 and causing apoptosis [59]. In this study, the oxidative stress and apoptosis indices of the si*MT* group were significantly increased, indicating that the degree of oxidative damage and apoptosis was more severe in the si*MT* group, which further proved that *Pe*MT plays an important role in antioxidation and anti-apoptosis.

### 3.4. Regulation Effect of PeMTF1 on PeMT

To explore the regulatory effects of *Pe*MTF1 on *Pe*MT, we performed RNAi experiments at the individual level. After RNAi with *PeMTF1* and 24 h of Zn stress, the expression level of *PeMT* significantly decreased (*p* < 0.05). Similarly, Meng et al. [60] found that the expression levels of *CgMT1* and *CgMT4* in the gills of *C. gigas* were significantly decreased after RNAi with *CgMTF1* and 48 h of Zn stress. Ren et al. [37] found that the expression level of *OcMT* in *O. chinensis* significantly decreased after RNAi with *OcMTF1.* Troadec et al. [61] knocked down *MTF1* in NIH 3T3 cells and found that the expression level of *MT* was significantly decreased. Moreover, Atanesyan et al. [62] found that the *MTnE* of *Drosophila* was almost unexpressed after RNAi with *MTF1* and was highly expressed after the overexpression of *MTF1*. These findings suggest that *Pe*MTF1 has a conserved function and participates in the regulation of *Pe*MT. *Pe*MT and *Pe*MTF1 are key factors allowing *P. esculenta* to endure the toxicity of heavy metal pollution.

## 4. Materials and Methods

### 4.1. Animals

*P. esculenta* individuals (3.9 ± 0.5) g were collected from Xizhou of Ningbo, Zhejiang, China. They were then kept in filtered seawater at a temperature of 20.5 ± 0.5 °C, salinity of 25.5 ± 0.5‰, and pH of 7.6 ± 0.2 for four days.

### 4.2. Treatments and Sampling

Preliminary experiments showed that the 96 h median lethal concentration (LC_50_) of Zn^2+^ for *P. esculenta* was 167.909 mg/L. Based on this value, four Zn^2+^ concentrations (0, 16.8, 33.6, and 84 mg/L) were set for 96 h of stress. Three tanks were used for each concentration, and 30 individuals were placed in each tank. The seawater was changed daily, and the Zn concentrations were kept constant.

Samples of the coelom fluid, intestine, constrictor muscle, nephridium, and body wall were collected from six individuals in the control group at 0 h, and the intestines of six individuals at each concentration were sampled after 6, 12, 24, 48, and 96 h of Zn stress. At each time point, two individuals were randomly selected from each tank, they were then sampled and mixed into one sample. The coelom fluid was extracted with a 1 mL syringe. Then, *P. esculenta* individuals were placed on the ice, and the intestines, constrictor muscles, nephridia, and body walls were collected and washed with distilled water. Finally, the samples were immediately frozen in liquid nitrogen and then stored at −80 °C. All experimental procedures were approved by the Animal Care and Use Committee of Ningbo University (Ningbo, China).

### 4.3. RNA Extraction and cDNA Synthesis

The RNA-Solv Reagent (Omega, GA, USA) was used to extract total RNA from the tissues of *P. esculenta*. Some of the obtained RNA was reverse transcribed by PrimeScript^®^ RT Kit (Takara, Dalian, China) for intermediate fragment cloning and tissue expression analysis, and the remaining was reverse transcribed by SMARTer RACE 5′/3′ reagent (Takara, Dalian, China) for 5′ and 3′ RACE (rapid amplification of cDNA ends).

### 4.4. Full-Length cDNA Cloning of PeMT and PeMTF1

We obtained the ORFs of *PeMT* and *PeMTF1* from the transcriptome data of *P. esculenta* (GenBank Accession No. OL757513) and designed specific primers using Primer Premier 5.0 for verification. Based on the verification results, specific primers for 5′ and 3′ RACE were designed, and full-length cloning was carried out. The primers used are listed in Table S1.

The PCR procedures involved the following steps: 94 °C 5 min; 8 cycles of 94 °C 30 s, 57 °C 30 s (with a decrease of 0.5 °C/cycle), and 72 °C 30 s; 27 cycles of 94 °C 30 s; 53 °C 30 s, and 72 °C 30 s; 72 °C 10 min. The product was separated by 1% agarose gel electrophoresis and purified by DNA Gel Extraction Kit (BioTeke, Beijing, China). Next, the purified product was ligated to the pMD-19T vector (Takara, Dalian, China) and transformed into Trans5α (TransGen Biotech, Beijing, China). Finally, positive clones were identified and sequenced by Zhejiang Youkang Biotechnology Co., Ltd. (Youkang, Zhejiang, China).

### 4.5. Bioinformatics Analysis of PeMT and PeMTF1

The ORFs of *PeMT* and *PeMTF1* were predicted by NCBI’s ORFfinder tool (https://www.ncbi.nlm.nih.gov/orffinder/, accessed on 16 June 2023). The sequences in the ORFs of *PeMT* and *PeMTF1* were translated into amino acids using the Bioxm 2.6 software. The molecular weights and isoelectric points of *Pe*MT and *Pe*MTF1 were predicted by the Expasy ProtParam tool (https://web.expasy.org/protparam/, accessed on 8 July 2023). The intracellular localization of *Pe*MTF1 was predicted by Cell-PLoc 2.0 (http://www.csbio.sjtu.edu.cn/bioinf/Cell-PLoc-2/, accessed on 20 June 2024). The DNA-binding domain of PeMTF1 was predicted by Smart (http://smart.embl-heidelberg.de/, accessed on 20 June 2024). The tertiary structures of *Pe*MT and *Pe*MTF1 were predicted by the I-TASSER (https://zhanglab.ccmb.med.umich.edu/I-TASSER/, accessed on 8 July 2023). The multiple sequence alignment and phylogenetic tree analysis of the MT and MTF1 homologs were performed by Vector NTI 11.5 and MEGA 5.1 software. The species and their GenBank Accession numbers used in the multiple sequence alignment and phylogenetic tree analysis are listed in Tables S2 and S3.

### 4.6. Tissue-Specific Expression of PeMT and PeMTF1 mRNA and Its Expression Changes under Zn Stress

Tissue-specific expression of *PeMT* and *PeMTF1* mRNA in the coelom fluid, intestine, constrictor muscle, nephridium, and body wall was monitored by RT-PCR, with the *β-actin* serving as an internal reference. The PCR procedures were as follows: 94 °C 5 min; 30 cycles of 94 °C 15 s, 55 °C 15 s, and 72 °C 15 s; 72 °C 5 min. The PCR products were separated by 1% agarose gel electrophoresis, visualized by a gel image analysis system (FR, Shanghai, China), and then analyzed by Image J software. All data are presented as mean ± SD (*n* = 3).

RT-qPCR was conducted to study the temporal expression profiles of intestinal *PeMT* and *PeMTF1* mRNA under Zn stress. The PCR procedures were as follows: 95 °C 5 min; 40 cycles of 95 °C 30 s, 55 °C 30 s, and 72 °C 30 s; 72 °C 5 min. Finally, the relative expression level of *PeMT* and *PeMTF1* mRNA was calculated by the 2^−ΔΔCT^ method. All data are presented as mean ± SD (*n* = 3). The primers used are listed in Table S1.

### 4.7. Expression and Purification of the Recombinant pGEX-6p-1-MT

Primers with restriction sites (*BamHI* and *XhoI*) were used to amplify the ORF of *PeMT*. The PCR product and pGEX-6p-1 vector were double digested with *BamHI* and *XhoI* restriction enzymes (Thermo Fisher Scientific, Waltham, MA, USA) and then ligated by T4 ligase (Takara, Dalian, China). The recombinant plasmid was transformed into Trans5α (TransGen Biotech, Beijing, China) and cultured overnight on a solid LB medium with 100 μg/mL of Ampicillin. The following day, positive clones were screened, and plasmids were extracted by the Plasmid Extraction Mini Kit (Solarbio, Beijing, China).

After sequencing, the correct recombinant plasmid was transformed into Transetta (DE3) (TransGen Biotech, Beijing, China) and cultured in the liquid LB medium with 100 μg/mL Ampicillin at 37 °C and 180 rpm. When the OD_600_ reached a density of 0.5–0.7, 1 mM of IPTG was added to induce the expression of the recombinant protein. Based on the SDS-PAGE analysis, the supernatant was purified by the GST-tag Protein Purification Kit (Beyotime, Shanghai, China).

### 4.8. Zn Tolerance of the Recombinant E. coli

The recombinant *E. coli* strains that transformed pGEX-6p-1-MT and pGEX-6p-1 were cultured in liquid LB medium with 100 μg/mL Ampicillin until the OD_600_ reached 0.4, and then 1 mM of IPTG was added. They were then divided into control and 0.3 mM Zn stress groups based on our pre-experiment (Figure S1), and their OD_600_ was measured in the following 8 h. The results are presented as mean ± SD (*n* = 3).

### 4.9. Antioxidant Capacity of the Recombinant pGEX-6p-1-MT

The pGEX-6p-1 protein was purified as described previously. We determined the concentrations of pGEX-6p-1 and pGEX-6p-1-MT using the Bradford Protein Concentration Assay Kit (Beyotime, Shanghai, China) and prepared 1 mg/mL of GSH. Then, they were diluted to 0.12, 0.18, 0.24, and 0.3 mg/mL, and the ABTS free radical scavenging ability was determined by the Total Antioxidant Capacity Assay Kit (Beyotime, Shanghai, China).

### 4.10. Detection of Intestinal Oxidative Stress and Apoptosis Indices after RNAi with PeMT and Zn Stress

Interference primers were designed and synthesized by Shanghai GenePharma Technology Co., Ltd. (GenePharma, Shanghai, China) (Table S1). Eight *P. esculenta* individuals (3.9 ± 0.5 g) were divided into siNC and si*MT* groups. Lipo6000^TM^ (Beyotime, Shanghai, China) and siRNA were diluted with 1 × PBS at a volume ratio of 17:3 and mixed at a volume ratio of 1:1. After mixing, the mixture was stored at room temperature for 5 min and injected at a dose of 1 μg/g. Then, *P. esculenta* individuals were exposed to 33.6 mg/L Zn for 24 h. The intestines were then collected and stored at −80 °C.

The interference efficiency of *PeMT* mRNA was determined by RT-qPCR using the method described above. Intestines were accurately weighed and mixed with extract solution in the ratio of weight (g)/volume (mL) = 1:10. The mixture was then homogenized in an ice bath and centrifuged at 10,000× *g* for 8 min at 4 °C. The oxidative stress indices (MDA, SOD, and GSH) and apoptosis indices (Caspase 3, Caspase 8, and Caspase 9) were measured by assay kits according to the instructions (Solarbio, Beijing, China).

### 4.11. Detection of Intestinal PeMT mRNA Expression Level after RNAi with PeMTF1 and Zn Stress

The RNAi method for *PeMTF1* was similar to that for *PeMT*. After sampling, the interference efficiency of *PeMTF1* mRNA and changes in the expression of *PeMT* mRNA were detected by RT-qPCR, as described above.

### 4.12. Statistical Analysis

SPSS 26.0, Excel 2016, and GraphPad Prism 8.0 were used for statistical analysis, and significance was tested using one-way ANOVA followed by Duncan’s test and independent samples *t*-test. Different letters simultaneously indicate a significant difference among the groups (*p* < 0.05).

## 5. Conclusions

*PeMT* and *PeMTF1* were cloned and identified from *P. esculenta*. Their expression patterns indicate that they respond rapidly to Zn stress. Prokaryotic expression, biochemical detection, and RNAi experiments provided evidence for the functions of *Pe*MT and *Pe*MTF1, suggesting that *Pe*MT plays an important role in Zn detoxification, antioxidation, and anti-apoptosis, and its expression is regulated by *Pe*MTF1, proving that *Pe*MT and *Pe*MTF1 are key factors allowing *P. esculenta* to endure the toxicity of Zn. This study made up for the gap in the functional research of MT and MTF1 in Sipuncula and accumulated basic data for studying the molecular toxicology of *P. esculenta*.

## Data Availability

Data are contained within the article and Supplementary Materials.

## Supplementary Materials

The following supporting information can be downloaded at: https://www.mdpi.com/article/10.3390/ijms25137368/s1.
