# Supplementary material for: Expression and Functional Analysis of the Metallothionein and Metal-Responsive Transcription Factor 1 in Phascolosoma esculenta under Zn Stress
Source: Int J Mol Sci. 2024 Jul 5;25(13):7368. doi: 10.3390/ijms25137368 (PMC11242308; doi:10.3390/ijms25137368)
Supplement: Supplementary file 1 [file ijms-25-07368-s001.zip › ijms-3074374-supplementary.pdf]

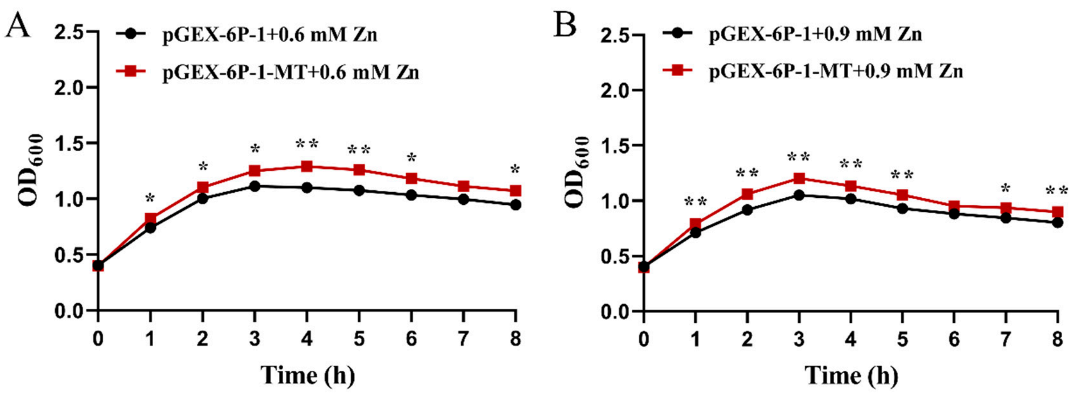

Figure S1. The growth status of the bacteria that transformed pGEX-6p-1-MT and pGEX-6p-1: (A) the 0.6 mM Zn stress group; (B) 0.9 mM Zn stress group. All data are presented as the mean ± SD (n = 3). “*”: $p < 0.05$; “**”: $p < 0.01$.

Table S1. The primers and their sequences

Primer	Sequence(5'-3')	Purpose
MT-F1	CCTTCAGTCTGCTGGTCTCT	PCR
MT-R1	TCTCGTTTTAGTGGCAGTCG	PCR
MTF1-F1	TAACTGTGATGAGAGCGGG	PCR
MTF1-R1	GCTGAAGTATCCACAAGACC	PCR
5’*MT*-R1	ATGCACTTCCCCGCTTTGGC	5’RACE
5’*MT*-R2	CCTTTGGTCCTCTGGGACTTCTGG	5’RACE
5’*MTF1*-R1	GTGTAACCTCTGGTGGGCTCGCAA	5’RACE
5’*MTF1*-R2	CACATCCCTGCACATCACACTCAAA	5’RACE
3’*MT*-F1	AAAGCGGGGAAGTGCAT	3’RACE
3’*MT*-F2	GTACCAAAGCTGGAAAATGC	3’RACE
3’*MTF1*-F1	GAGGCAGGTCGGGGCAGTGGTAG	3’RACE
3’*MTF1*-F2	CCTGTGATACAAACCGTGATTCCTGG	3’RACE
q*MT*-F	TGCTTTGAGACAAGATGCC	RT-PCR&RT-qPCR
q*MT*-R	ACTTCCCCGCTTTGACAC	RT-PCR&RT-qPCR
q*MTF1*-F	CAGTCTCAAGACTCACACAT	RT-PCR&RT-qPCR
q*MTF1*-R	TTGCTATGGCCATCTCTAAG	RT-PCR&RT-qPCR
β-actin-F	CACGGAATCGTCACCAACT	Reference gene
β-actin-R	AGAAGCATACAGGGACAGCA	Reference gene
yh-*MT*-F	CGC**GGATCC**ATGCCGGCAACTGACTGC	Prokaryotic expression
yh-*MT*-R	CCG**CTCGAG**CTTGCATTTGCAGCCCTC	Prokaryotic expression
si*MT*-sense	CCAGCUGUAAAUGCAGCAATT	RNA inference
si*MT*-antisense	UUGCUGCAUUUACAGCUGGTT	RNA inference
si*MTF1*-sense	GCCAGAUGGAGAGAAGUAUTT	RNA inference
si*MTF1*-antisense	AUACUUCUCUCCAUCUGGCTT	RNA inference
siNC-sense	UUCUCCGAACGUGUCACGUTT	RNA inference
siNC-antisense	ACGUGACACGUUCGGAGAATT	RNA inference

Table S2. The GenBank accession numbers and isforms of the MT homologs

Specie	GenBank accession number	Isoform
Sinohyriopsis cumingii	ACZ06027.1	MT
Alectryonella plicatula	ALA14799.1	MT
Magallana gigas	CAB64869.1	MT
Perinereis nuntia	AFE61618.1	MT
Pomacea canaliculata	XP_025111375.1	MT 20-III-like X3
Cerastoderma edule	CDF59687.1	MT
Perna viridis	AEX60795.1	MT1D
Danio rerio	NP_571150.1	MT1
Carassius auratus	AAB32777.1	MT
Oryzias latipes	NP_001098255.1	MT
Gallus gallus	BAF51974.1	MT
Homo sapiens	AAP97267.1	MT1M
Mus musculus	AAQ24163.1	MT1K
Zootoca vivipara	CAJ32319.1	MT

Note: For *Homo sapiens*, *Mus musculus* and other species with multiple isoforms, wo compared their similarities and finally chose MT or MT1 for comparison.

Table S3. The GenBank accession numbers and isoforms of the MTF1 homologs

Specie	GenBank accession number	Isoform
Chaetura pelagica	XM_009996683.1	MTF1
Crotalus tigris	XM_039329224.1	MTF1
Lacerta agilis	XM_033157482.1	MTF1
Mus musculus	NP_032662.3	MTF1
Rattus norvegicus	NP_001102147.1	MTF1
Homo sapiens	NP_005946.2	MTF1
Gallus gallus	NP_001026666.1	MTF1
Xenopus tropicalis	XP_031752770.1	MTF1
Rana temporaria	XP_040193139.1	MTF1
Danio rerio	NP_694513.1	MTF1
Oryzias melastigma	XP_036071630.1	MTF1
Strongylocentrotus purpuratus	XP_030850345.1	MTF1
Mytilus edulis	CAG2198514.1	MTF1
Mytilus coruscus	CAC5383271.1	MTF1
Chionoecetes opilio	KAG0726832.1	MTF1
